# A CRISPR-based base-editing screen for the functional assessment of BRCA1 variants

**DOI:** 10.1038/s41388-019-0968-2

**Published:** 2019-08-29

**Authors:** Jiyeon Kweon, An-Hee Jang, Ha Rim Shin, Ji-Eun See, Woochang Lee, Jong Won Lee, Suhwan Chang, Kyunggon Kim, Yongsub Kim

**Affiliations:** 1 Department of Biomedical Sciences, Asan Medical Institute of Convergence Science and Technology, Asan Medical Center, University of Ulsan College of Medicine, Seoul, Republic of Korea; 2 Stem Cell Immunomodulation Research Center, University of Ulsan College of Medicine, Seoul, Republic of Korea; 3 Department of Laboratory Medicine, Asan Medical Center, University of Ulsan College of Medicine, Seoul, Republic of Korea; 4 Division of Breast Surgery, Department of Surgery, Asan Medical Center, University of Ulsan College of Medicine, Seoul, Republic of Korea; 5 Department of Convergence Medicine, Asan Medical Center, University of Ulsan College of Medicine, Seoul, Republic of Korea; 6 Clinical Proteomics Core Lab, Asan Institute for Life Sciences, Seoul, Republic of Korea

**Keywords:** Breast cancer, Biological techniques

## Abstract

Genetic mutations in *BRCA1*, which is crucial for the process of DNA repair and maintenance of genomic integrity, are known to increase markedly the risk of breast and ovarian cancers. Clinical genetic testing has been used to identify new *BRCA1* variants; however, functional assessment and determination of their pathogenicity still poses challenges for clinical management. Here, we describe that CRISPR-mediated cytosine base editor, known as BE3, can be used for the functional analysis of *BRCA1* variants. We performed CRISPR-mediated base-editing screening using 745 gRNAs targeting all exons in *BRCA1* to identify loss-of-function variants and identified variants whose function has heretofore remained unknown, such as c.-97C>T, c.154C>T, c.3847C>T, c.5056C>T, and c.4986+5G>A. Our results show that CRISPR-mediated base editor is a powerful tool for the reclassification of variants of uncertain significance (VUSs) in *BRCA1*.

## Introduction

The breast cancer type 1 susceptibility gene (*BRCA1*) is a tumor suppressor gene related to the maintenance of genome integrity [1]. Inherited loss-of-function (LOF) mutations of *BRCA1* confer susceptibility to breast, ovarian, prostate, and pancreatic cancer; therefore, the identification and functional assessment of *BRCA1* variants is important for the clinical management of various diseases [2]. Advances in sequencing technology led to the identification of many *BRCA1* variants through clinical genetic testing. To investigate the pathogenicity of these *BRCA1* variants, various functional assessment methods have been developed, including fluorescent reporter assays, embryonic stem cell viability assays, and therapeutic drug-based sensitivity assays [3]. These assays utilize exogenously expressed *BRCA1* variants and have clarified the function of a lot of *BRCA1* variants, however, the exogenous expression often result in their overexpression, which can affects gene dosage, protein folding, complex assembly, and downstream regulation. Furthermore, these assays cannot be applied to the posttranscriptional regulation such as mRNA splicing, transcript stability, and effect of untranslated region [4].

Engineered nucleases, represented by the CRISPR-Cas9 system, were developed for targeted genome editing in living cells and organisms [5]. The CRISPR-Cas9 system induces chromosomal DNA double-strand breaks in a target sequence-specific manner, which are repaired via error-prone nonhomologous end joining or error-free homology-directed repair (HDR), resulting in gene disruption, addition, and correction. CRISPR-mediated HDR can be used to introduce point mutations; however, it typically induces unwanted insertion and deletion (indel) mutations. In addition, because the system shows low efficiency and requires homologous DNA templates, it is inadequate for introducing various mutations into large genes such as *BRCA1* or *BRCA2*.

Recently, several groups have shown that Cas9 nickase or catalytically inactive Cas9 (dead Cas9, dCas9) fused with cytidine deaminase induces target-specific nucleotide substitutions in live cells and organisms in the absence of homologous DNA templates [6–9].

Here, we used the Base Editor 3 (BE3), which induces targeted C:G to T:A conversions, for the functional assessment of *BRCA1* variants, and identified the pathogenicity of *BRCA1* variants with unknown functions through BE3-mediated high-throughput screens.

## Results

**Fig. 1:**
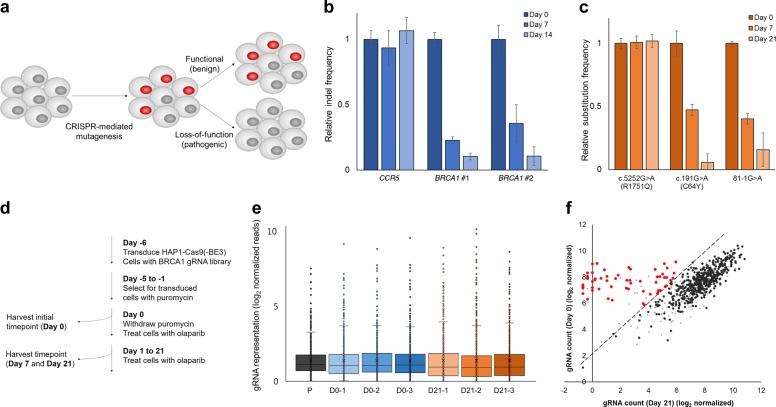
Functional assessment of *BRCA1* variants using CRISPR-based base editing. **a** Schematic overview of the functional analysis of *BRCA1* via targeted mutagenesis. **b** Cell viability analysis of HAP1-Cas9 cells transfected with two different gRNAs targeting *BRCA1* using targeted deep sequencing. *BRCA1* #1 and *BRCA1* #2 indicate each *BRCA1*-targeting gRNA, and the *CCR5*-targeting gRNA was used as a negative control. **c** Cell viability analysis of HAP1-BE3 cells transfected with gRNAs targeting pathogenic mutations [c.81-1G>A and c.191G>A (p.C64Y)] and a benign mutation [c.5252G>A (p.R1751Q)] using targeted deep sequencing. **d** Timeline of *BRCA1* variant screens in HAP1-Cas9 and -BE3 cells. **e** Box plot showing the distribution of gRNA frequencies at different time points after gRNA transduction. **f** Scatterplot showing the depletion of specific gRNAs after 21 days. Error bars show the standard error of the mean

Because *BRCA1* plays an important role in the process of HDR, LOF of *BRCA1* affects cell viability, and this property can be used to evaluate the function of *BRCA1*. In other words, the introduction of *BRCA1* variants with LOF mutations into a cell results in cell death with increasing passage numbers, and this can be detected through analysis of mutation frequencies (Fig. 1a).

The HAP1 cell is widely used in genetic screens, because it is nearly-haploid cell lines, and LOF can be effectively introduced into the cells [10, 11]. We, first, examined whether functional assessment of *BRCA1* is possible in the HAP1 cell line. We generated Cas9-expressing HAP1 (HAP1-Cas9) and BE3-expressing HAP1 (HAP1-BE3) cell lines by infecting HAP1 cell lines with lentiviral particles expressing Cas9 or BE3. The genome editing activities of single clones were then analyzed to select highly active clones. The clones HAP1-Cas9 #7 and HAP1-BE3 #5 were chosen for further study (Supplementary Fig. 1).

As a proof-of-concept, *BRCA1*-targeting gRNAs were transfected into HAP1-Cas9 cell lines to disrupt BRCA1 and the cells were cultured for 14 days with 0.5 µM olaparib, inhibitor of poly ADP ribose polymerase (PARP), which increases the sensitivity of BRCA1 deficiency [12]. The mutation frequencies were measured by targeted deep sequencing, and the relative indel frequencies were significantly decreased with time (Fig. 1b).

HAP1-BE3 cell lines were used to induce pathogenic mutations disrupting *BRCA1* function by nucleotide substitutions. Three different mutations (c.81-1G>A, c.191G>A, and c.5252G>A) were introduced into HAP1-BE3 cells, and the cells were cultured for 14 days with 0.5 µM olaparib [13] (Fig. 1c). The relative substitution frequencies of c.81-1G>A and c.191G>A (p.C64Y) *BRCA1* mutations, known pathogenic variants, were decreased, whereas that of the likely benign missense variant, c.5252G>A (p.R1751Q), was retained. These results indicated that functional assessment of *BRCA1* can be performed in HAP1 cell lines, as reported previously [14], and HAP1-BE3 cell lines can be useful for introducing desired mutations into endogenous *BRCA1*.

Next, we designed a pooled gRNA library targeting *BRCA1* to perform CRISPR-based high-throughput screens. We selected 745 gRNAs that could target all exon sequences and 15 bp sequences around the exon–intron junction. Among the 745 gRNAs, 533 gRNAs can induce C:G to T:A conversions and a total of 660 C:G to T:A conversions can be generated by 533 gRNAs in *BRCA1*. These gRNAs were synthesized as pooled oligonucleotides and subcloned into lentiviral vectors via isothermal assembly. To determine whether the relative abundance of gRNAs could be used as a readout for *BRCA1* functional assessments, as in most CRISPR-based high-throughput screens [15], we transduced gRNAs capable of introducing pathogenic mutations into *BRCA1*, and both the relative abundance of gRNAs and endogenous mutations were analyzed. For the high-throughput functional assessment of *BRCA1*, the pooled gRNA library was transduced into HAP1-Cas9 or HAP1-BE3 cells with an MOI of 0.3, and the infected cells were cultured for 21 days with 0.5 µM olaparib, a PARP inhibitor with synthetic lethality in *BRCA1*-deficient cells (Fig. 1d). The change in the relative abundance of gRNAs was analyzed by targeted deep sequencing of three biological triplicates of genomic DNA.

The relative abundance of 24.2% of gRNAs (180 of the 745 gRNAs) in HAP1-Cas9 cells and 8.1% of gRNAs (60 of the 745 gRNAs) in HAP1-BE3 cells decreased after 21 days (fold change >4; *p* < 0.05; FDR < 0.25) (Fig. 1e, f and Supplementary Fig. 2). Unlike HAP1-Cas9 cells, which disrupt *BRCA1* by frameshift mutations, HAP1-BE3 cells induced LOF of *BRCA1* through nucleotide substitution, and relatively few gRNAs were depleted. We analyzed the potential off-target effect of each of 60 gRNAs using Cas-OFFinder and excluded gRNAs targeting more than three loci, resulting in the identification of 27 gRNAs as candidates for the induction of *BRCA1* dysfunction (Supplementary Table 1) [16]. The target positions of the 27 gRNAs were not limited to specific exons but were distributed among various exons of *BRCA1* (Supplementary Fig. 3). The *BRCA1* variants that could be induced by each of the 27 gRNAs were identified in the ClinVar database, which showed that 13 gRNAs introduced known pathogenic mutations in *BRCA1* [13].

**Fig. 2:**
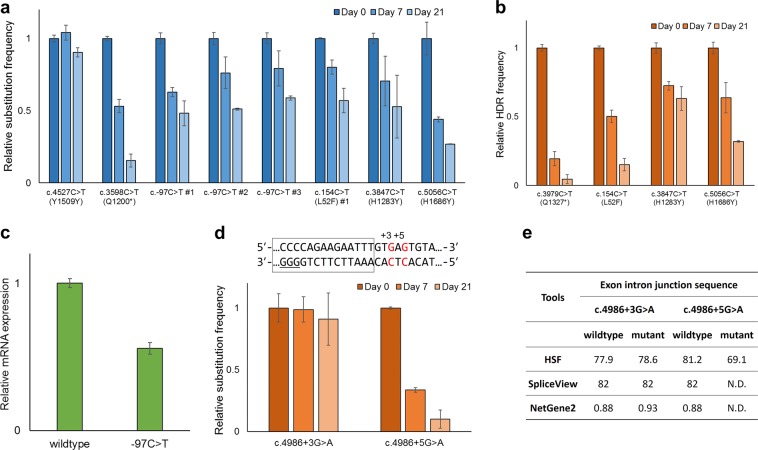
Validation of individual *BRCA1* variants inducing *BRCA1* dysfunction. **a** Cell viability analysis of HAP1-BE3 cells transfected with each candidate gRNA using targeted deep sequencing. The gRNAs inducing c.4527C>T and c.3598C>T were used as a negative and a positive control, respectively. **b** Functional validation of *BRCA1* variants using the CRISPR-based HDR method. **c** 5′-UTR reporter assays confirming the transcriptional repression of *BRCA1* by the −97C>T mutation. **d** Cell viability analysis of the intronic mutations c.4986+3G>A and c.4986+5G>A induced by a single gRNA. Targetable C:G pairs and PAM are in red and underlined, respectively. Exon is shown as a rectangle. **e** In silico analysis of c.4986+3G>A and c.4986+5G>A using Human Splicing Finder, SpliceView, and NetGene2. Duplicate wells for each gRNA at each time point were processed. Error bars show the standard error of the mean

Based on the results of high-throughput screens, we selected six gRNAs for further functional assessment of each BRCA1 variant; three gRNAs for c.−97C>T, one gRNA each for c.154C>T, c.3847C>T, and c.5056C>T. The c.3598C>T and c.4527C>T inducing gRNAs were used as positive and negative controls, respectively. Each gRNA was cloned into a plasmid DNA vector and transfected into HAP1-BE3 cells, and the endogenous *BRCA1* mutations induced by the gRNAs were tracked by targeted deep sequencing for 21 days (Fig. 2a and Supplementary Fig. 4). The relative mutation frequencies of c.3598C>T (p.Q1200*), a pathogenic variant, dramatically decreased, whereas those of c.4527C>T (p.Y1509Y), the benign variant, remained similar. We analyzed three *BRCA1* variants, c.154C>T (L52F) in the RING domain, c.3847C>T (H1283Y) in the SQ/TQ cluster domain, and c.5056C>T (H1686Y) in the BRCT domain, which are reported as variants of uncertain significance (VUSs) in the ClinVar database. The relative mutation frequencies of the three variants were decreased in a time-dependent manner, and the function of c.154C>T (L52F), c.3847C>T (H1283Y), and c.5056C>T (H1686Y) was verified by CRISPR-mediated HDR (Fig. 2b). The results suggested that these three VUSs affected *BRCA1* function and could be categorized as pathogenic mutations. Analysis of the c.−97C>T variant in the 5′-UTR region, which could be induced by three different gRNAs, showed that the relative mutation frequencies were significantly reduced in cells transfected with the three gRNAs. The 5′-UTR region of *BRCA1* might regulate the transcription level, and several mutations in the 5′-UTR region are known as pathogenic variants. To further validate the c.−97C>T variant, we performed a luciferase reporter assay in HEK293T/17 cells, which showed that the c.−97C>T mutation in the 5′-UTR caused a twofold downregulation of gene expression (Fig. 2c). This led to the identification of a novel potentially pathogenic mutation in the 5′-UTR region of *BRCA1* and suggested the importance of the UTR region for clinical genetic testing. We additionally confirmed the c.4986+3G>A and c.4986+5G>A variants induced by a gRNA targeting the splicing junction. As shown in Fig. 2d, the relative mutation frequencies of the c.4986+5G>A variant decreased with time, whereas those of the c.4986+3G>A variant remained similar. In silico analysis showed that only c.4986+5G>A, but not c.4986+3G>A, disrupted the splicing donor site; therefore, the c.4986+5G>A variant, which was previously reported as a VUS, could be classified as a pathogenic variant (Fig. 2e) [17, 18].

## Discussion

In summary, this paper describes the first application of the BE3 system for the functional assessment of *BRCA1* and the successful development of a high-throughput CRISPR-mediated base-editing screen for the identification of LOF variants in *BRCA1*. CRISPR-mediated base-editing screens identified several VUSs, including c.154C>T (L52F), c.3847C>T (H1283Y), c.5056C>T (H1686Y), and c.4986+5G>A, that disrupt the function of *BRCA1*, and these variants might be classified as pathogenic mutations.

Komor et al. developed BE3 and demonstrated that it could be used to introduce several disease-relevant mutations into mammalian cells [6]. We performed high-throughput functional assessment of *BRCA1* using the BE3 system and determined the lethality of dysfunctions in *BRCA1*, which is an approach that eliminates the need to create individual mutant cell lines. Findlay et al. recently developed a saturation genome editing (SGE) method based on CRISPR-mediated HDR and used it for the functional assessment of ~4000 single nucleotide variants (SNVs) in 13 small exons of *BRCA1* [14, 19]. Compared to our base-editing screens, the SGE method has no limitation regarding the target positions of SNVs because of the use of an artificial homologous donor DNA template. However, the SGE method is based on CRISPR-mediated HDR, and it is therefore difficult to use for screening large genes because of limitations in the production of donor DNA template. Although the base editor is limited regarding the gRNA design and C:G-to-T:A conversion, the development of the CRISPR-Cas system (e.g., CRISPR-based adenosine base editing [20], Cas variants with altered PAM sequences [5, 21, 22]) extends the use of base-editing screens for biomedical research.

## Methods

### Construction of plasmid DNA

spCas9 in lentiCas9-Blast (Addgene plasmid #52962) and BE3 in pCMV-BE3 (Addgene plasmid #73021) were used for genome editing. To construct the lentiBE3-Blast plasmid DNA for lentivirus production, the BE3 coding regions were amplified by PCR and cloned into the lentiCas9-Blast vector using XbaI (NEB #R0145) and BamHI (NEB #R3136) restriction enzymes. The gRNA constructs were cloned into lentiGuide-puro (Addgene plasmid #52963), and the target sequences of each gRNA are listed in Supplementary Table 2.

### Design and construction of gRNA library

To design *BRCA1*-targeting gRNAs, exon sequences and the 15 bp regions around the sequences of exon–intron junctions were obtained from GenBank at NCBI [23]. Then, all possible target sites with 5′-(N)_x20_-NGG-3’ and 5′-CCN-(N)_x20_-3′ were searched using Cas-Designer (http://www.rgenome.net/cas-designer/) and 745 gRNAs-targeting *BRCA1* were listed. To generate a gRNA library, pooled oligonucleotides containing the coding sequences of the gRNAs were synthesized (Custom Array Inc.) and cloned into the lentiGuide-puro vector as previously described [24].

### Cell culture and construction of Cas9- or BE3-expressing cell lines

HAP1 cells were maintained in Iscove’s modified Dulbecco’s medium with 10% fetal bovine serum (FBS) and 1% penicillin/streptomycin at 37 °C and 5% CO_2_, and HEK293T/17 cells were maintained in Dulbecco’s modified Eagle’s medium with 10% FBS and 1% penicillin/streptomycin at 37 °C and 5% CO_2_. To generate HAP1-Cas9 and HAP1-BE3 cells, lentiviral particles were generated with 15 μg of lentiCas9-Blast (or lentiBE3-Blast) and two viral packaging plasmids (9 μg of psPAX2 and 6 μg of pMD2.G) as previously described and transduced into HAP1 cells with an MOI of 0.1 [25]. The infected HAP1 cells were selected on media containing 10 μg/mL blasticidin, and single clones were isolated. To select HAP1-Cas9 and HAP1-BE3 single clones, each of the seven clones of HAP1-Cas9 and HAP1-BE3 were infected with CCR5 targeting gRNAs, and mutation frequencies were analyzed by the T7 endonuclease I (T7E1) assay and targeted deep sequencing as previously described [26]. Among them, the highly active single clones (HAP1-Cas9 #7 and HAP1-BE3 #5) were selected and used in this study (Supplementary Fig. 1)

### Base-editing screen and analysis

For gRNA library screening, 2 × 10^6^ HAP1-Cas9 or HAP1-BE3 cells were seeded into six-well plates, and lentiviral particles of the gRNA library were infected with an MOI of 0.3. The infected cells were selected on medium containing 1 μg/mL puromycin. After 7 days of puromycin selection, at least 5 × 10^5^ cells were collected to measure the frequency of each gRNA in the initial pool (Day 0), and 1 × 10^6^ cells were maintained with 0.5 μM olaparib. After 7 and 21 days, the cells were collected, and genomic DNA was isolated using the DNeasy Blood & Tissue Kit (Qiagen, Cat. No: 69504) for analysis by targeted deep sequencing as previously described. The screening data were analyzed using Count_space.py and the pipeline MAGeCK (ver. 0.5.6). In BE3-based screens, 212 gRNAs with no C in the targetable range of each gRNA were used as non-target controls. Positions of coding nucleotides and amino acids in *BRCA1* are referenced by ClinVar transcript annotation for BRCA1, transcript NM_007294.3 (NCBI).

### Targeted deep sequencing analysis

Twenty-four hours before transfection, 5 × 10^5^ HAP1(-Cas9 or -BE3) cells were seeded in 24-well plates or 1 × 10^5^ HAP1(-Cas9 or -BE3) cells were seeded in 96-well plates (Corning). All transfection experiments were conducted using FuGENE HD (Promega, Cat. No: E2311) according to the manufacturer’s protocol. Three days after transfection, genomic DNAs were isolated from each well using the DNeasy Blood & Tissue Kit (Qiagen) and used as the Day 0 sample. To analyze endogenous mutation frequencies, the target regions in genomic DNAs were amplified with appropriate primers using Phusion DNA polymerase (New England Biolabs) according to the manufacturer’s protocol. The PCR amplicons were confirmed using 2% agarose gel electrophoresis and subjected to Illumina MiniSeq. The sequencing data were analyzed using Cas-Analyzer (http://www.rgenome.net/cas-analyzer/). The number of sequence reads are shown in Supplementary Table 3. The PCR primer sequences are listed in Supplementary Table 4.

### Luciferase assay

To construct the *BRCA1* 5′-UTR reporters, the 232 bp wild-type and variant sequences were cloned into pGL4.20 with the CMV promoter. The luciferase reporters were transfected into HEK293T/17 cells, and luciferase activities were measured with the Dual-Luciferase® Reporter Assay System (Promega Cat. No: E1960) according to the manufacturer’s protocol.

## Supplementary information

SI

## Data Availability

The deep sequencing data are available at the NCBI Sequence Read Archive (SRA) under accession number PRJNA529534.
