# Supplementary material for: A CRISPR-based base-editing screen for the functional assessment of BRCA1 variants
Source: Oncogene. 2019 Aug 29;39(1):30–5. doi: 10.1038/s41388-019-0968-2 (PMC6937211; doi:10.1038/s41388-019-0968-2)
Supplement: Supplementary file 1 — SI [file 41388_2019_968_MOESM1_ESM.docx]

**Supplementary Information**

**A CRISPR-based base-editing screen for the functional assessment of BRCA1 variants**

Jiyeon Kweon^1,2^, An-Hee Jang^1,2^, Ha Rim Shin^1,2^, Ji-Eun See^1,2^, Woochang Lee^3^, Jong Won Lee^4^, Suhwan Chang^1^, Kyunggon Kim ^5,6^, Yongsub Kim^1,2,*^

**Figure 1.** Generation of Cas9 or BE3 expressing HAP1 cell line.

**Figure 2.** *BRCA1* screen in HAP1-Cas9

**Figure 3.** Distribution of gRNAs selected as candidates to induce nucleotide substitutions.

**Figure 4.** Cell viability analysis of HAP1-BE3 cells transfected with other gRNA inducing c.154C>T (L52F) mutation.

**Table 1.** gRNA candidate in *BRCA1* variants screen.

**Table 2.** List of target sequences of each gRNA used in this study.

**Table 3.** The raw numbers of sequence reads in this study.

**Table 4.** List of oligonucleotides used in this study.

**
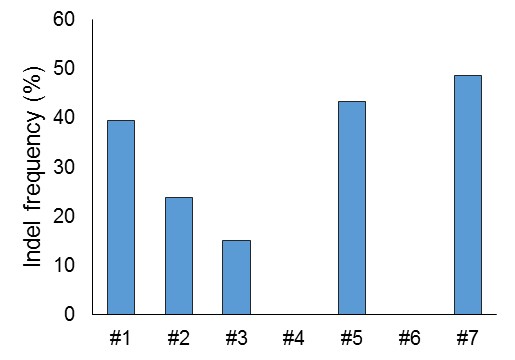
a**

**
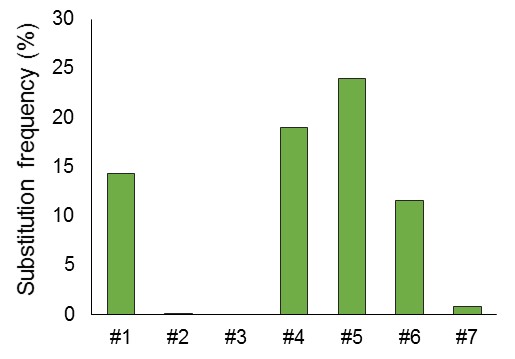
b**

**Figure 1.** Generation of Cas9 or BE3 expressing HAP1 cell line. (a) Mutation frequencies HAP1-Cas9 single clones were analyzed by T7E1 assay. HAP1-Cas9 #7 clone was used for further study. (b) Substitution frequencies HAP1-BE3 single clones were analyzed and by targeted deep sequencing and HAP1-BE3 #5 clone was used for further study.

**
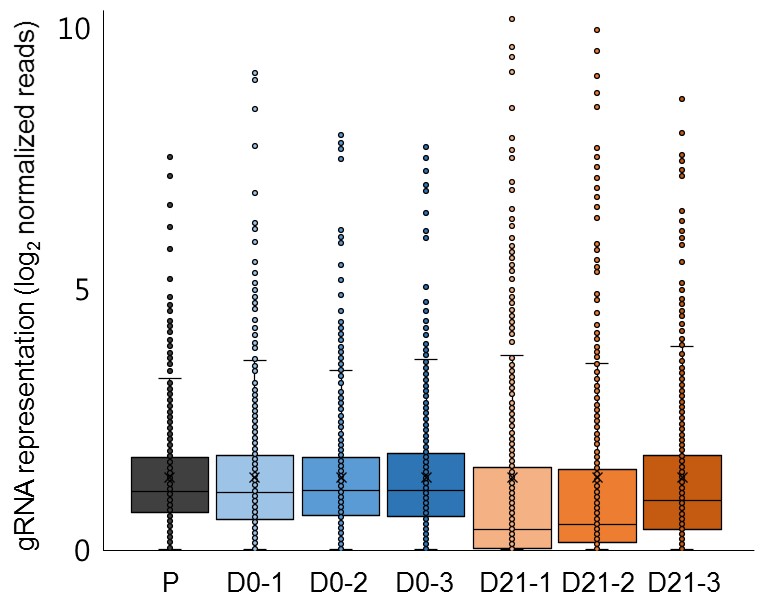
a**

**b**

**
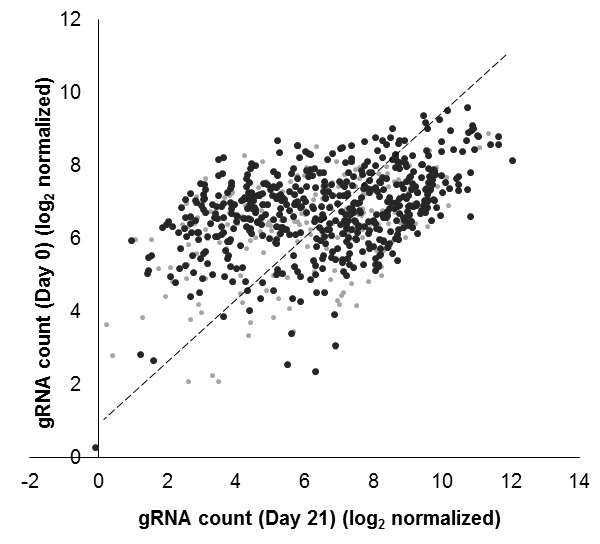
**

**Figure 2.** *BRCA1* screen in HAP1-Cas9 (a) Box plot showing the distribution of gRNA frequencies at different time points after gRNA transduction (b) Scatterplot showing the depletion of specific gRNAs after 21 days.

**
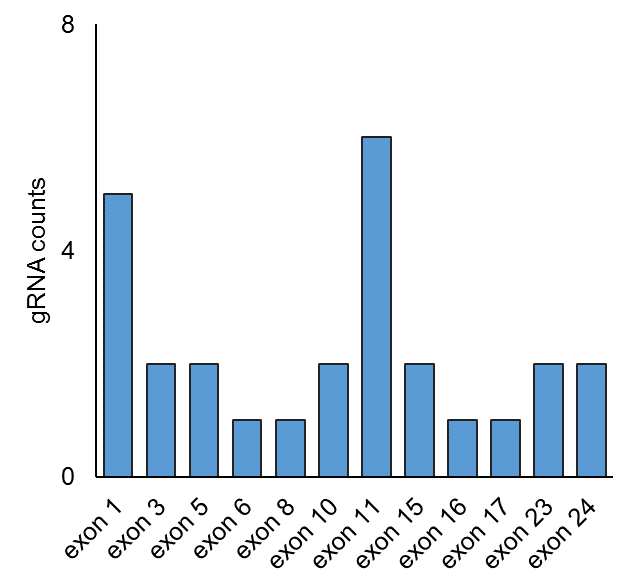
**

**Figure 3.** Distribution of gRNAs selected as candidates to induce nucleotide substitutions. The selected 27 gRNAs were distributed various exons including untranslated region.

**
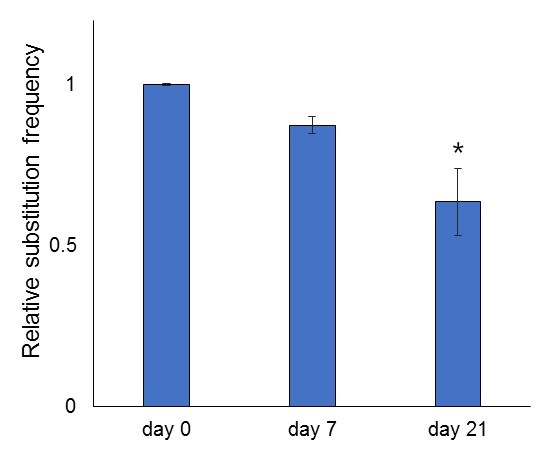
**

**Figure 4.** Cell viability analysis of HAP1-BE3 cells transfected with other gRNA inducing c.154C>T (L52F) mutation. **p*<0.05 compared with day 0.

**Table 1** gRNA candidate in *BRCA1* variants screen. Variants listed in ClinVar database are highlighted as pathogenic in red, benign in blue and VUS in green.

**Table 2.** List of target sequences of each gRNA used in this study.

**Table 3.** The raw numbers of sequence reads in this study.

**Table 4.** List of oligonucleotides used in this study
